# American Physician Scientist Association annual meetings: celebrating 20 years of physician-scientist training and collaboration

**DOI:** 10.1172/jci.insight.198595

**Published:** 2025-09-09

**Authors:** Cynthia Y. Tang, Alex D. Waldman, Daniel C. Brock

**Affiliations:** 1 University of North Carolina MD-PhD Program, University of North Carolina-Chapel Hill, Chapel Hill, North Carolina, USA.; 2 American Physician Scientists Association, Westford, Massachusetts, USA.; 3 Department of Neurosurgery, MedStar Georgetown University Hospital, Washington, DC, USA.; 4 Baylor College of Medicine Medical Scientist Training Program, Houston, Texas, USA.

## Abstract

The American Physician Scientists Association (APSA) was founded in 2003 with a mission to build a unified community for physician-scientist trainees. Over the past 2 decades, the APSA has played a pivotal role in fostering the development of future physician-scientists through mentorship, advocacy, and professional development. This year, the APSA hosted its 20th Annual Meeting in Chicago in collaboration with the Association of American Physicians and the American Society for Clinical Investigation. This milestone marks a moment of celebration and reflection, highlighting APSA’s enduring impact on the future of physician-scientist training.

Physician-scientists have a fundamental role in the advancement of basic, translational, and clinical discoveries. Their specialized training bridges both medical and scientific arenas, with the overall goal of translating biomedical innovations to improve patient care. However, the physician-scientist training pipeline is arduous and fraught with challenges to achieving success. In 2003, a group of students, led by Freddy Nguyen (1), recognized a critical gap in support for physician-scientist trainees. Motivated by the absence of a dedicated national organization for this unique cohort, Nguyen and fellow trainees created the American Physician Scientists Association (APSA). Their vision was to create a platform that would provide mentorship, advocacy, and community for physician-scientist trainees across the United States.

As part of this vision, the inaugural APSA Annual Meeting was held in April 2005 in Chicago in parallel with the Joint Meeting of the Association of American Physicians (AAP) and the American Society for Clinical Investigation (ASCI). This collaboration aimed to connect trainees with established physician-scientists. This first meeting included 55 APSA members who had the opportunity to present posters to AAP/ASCI attendees and participate in high-caliber scientific sessions, including presentations from Nobel laureates and leaders within biomedical research, setting a high bar for quality and value of future conferences. In the subsequent 20 years, the APSA has experienced substantial growth, evolving from a grassroots initiative to a nationally recognized nonprofit organization with international reach. In 2016, the APSA was formally integrated into what is now known as the AAP/ASCI/APSA Joint Meeting. APSA’s Joint Meeting partnerships have expanded to include financial support from the NIH, Lasker Foundation, Burroughs Wellcome Fund (BWF), and multiple specialty medical societies. These strong collaborations have been fundamental to APSA’s growth and ongoing success (2).

APSA’s membership has steadily increased over time, reflecting the growing demand for trainee-centric resources and support within the physician-scientist community. The organization’s commitment to fostering the next generation of physician-scientists is evident through its sustained efforts to provide mentorship, professional development opportunities, and advocacy for trainees at all career stages. Today, the APSA is the leading resource and community for physician-scientists in training in the United States and globally, serving over 2,200 members across more than 100 institutions.

The AAP/ASCI/APSA Joint Meeting has become the central source for physician-scientist trainees to create a peer community across institutions, identify mentors across the spectrum of specialties and career levels, explore career opportunities, and develop professional skills. As immediate past presidents (CYT, ADW) of the APSA, we reflect on the expansion of the APSA Annual Meeting over the past 20 years and discuss opportunities for continued growth, as we enter the next decade of the APSA.

## APSA annual meetings: 20 years of development, mentorship, and community

The AAP/ASCI/APSA Joint Meeting offers a diverse repertoire of programming that provides trainees with opportunities to network with peers and established physician-scientists, identify lifelong mentors, gain exposure to cutting-edge science, and access professional development sessions and resources. As the premier annual gathering of physician-scientists, this meeting features high-level innovations and transformative discoveries spanning the topics of science, clinical care, health care systems, and public policy from distinguished researchers across the academic, industry, and government sectors.

APSA’s programming during the Joint Meeting is meticulously curated with three primary goals in mind to meet the evolving needs of the community: (i) provide professional development opportunities for current and future physician-scientists, (ii) develop a robust and sustainable community of physician-scientists, and (iii) foster an accessible and sustainable pipeline for current and future trainees.

### Professional development.

Career development has always been a cornerstone of APSA’s programming. The inaugural APSA meeting in 2005 featured a panel titled “Navigating the MD/PhD Pathway,” and sessions focused on supporting trainees at various stages of the physician-scientist journey continue to be offered at the Joint Meeting. APSA sessions are intentionally developed to highlight the evolving biomedical landscape and unique career pathways for physician-scientists. Speakers span diverse fields and unique career trajectories across industry, academia, government, and nonprofit sectors, and sessions have included the following topics: “Building a Research Portfolio in Translational Medicine,” “Career Paths in Academic Medicine,” and “Career Development in Translational Medicine.” Given the increased interest of our membership in pursuing careers outside of academic medicine, the APSA hosted these recent sessions: “Innovations and Leadership in Biotechnology” in 2024 and “Career Opportunities in Biotech and Pharmaceuticals” in 2025.

In 2024, the APSA introduced a series titled “Interviewing an Icon,” sponsored by the Lasker Foundation, that features live interviews between APSA leaders and prominent physician-scientists as the first session of the Joint Meeting. Notable guests have included Betsy Nabel, MD, in 2024 and Peggy Hamburg, MD, in 2025, who both shared insights into their careers within science, medicine, leadership, and public service. These interactive sessions have added a personal and inspirational dimension to APSA’s longstanding commitment to career development.

In addition to these sessions, the APSA runs the highly revered Subspeciality-Interest Breakfast and Residency Luncheon, where trainees can connect and learn about physician-scientist careers within a diverse array of clinical fields and meet with residency program directors, respectively. These programs provide an opportunity for trainees to learn more about postgraduate training opportunities, and for programs to recruit the nation’s most promising students. In 2025, more than 20 subspecialties were represented at the breakfast, and a record number (over 30) of residency programs participated in the luncheon. Since 2022, the impact of these sessions has only continued to grow, particularly through collaboration with the ASCI/Alliance for Academic Internal Medicine/BWF Physician-Scientist Pathways Annual Workshop, which takes place in conjunction with these events.

The APSA also has a strong commitment to supporting undergraduate and postbaccalaureate trainees interested in entering the physician-scientist pipeline, as well as residents and fellows preparing to launch their careers. Specialized programming tailored to each of these groups has included sessions such as “Tips and Tricks for the Developing Pre-Trainee” and “Negotiating a Job Offer.”

### Community building.

Trainees have opportunities throughout the conference to network with peers and physician-scientists across all career stages. The Welcome Reception includes informal spaces for trainees to meet peers from across the United States and around the world. The Awards Dinner serves as a time to celebrate individuals who have made substantial contributions to the organization and to physician-scientist training. The poster sessions provide excellent forums for trainees to hone their research communication skills and network with AAP and ASCI members and other leaders in academia and industry. These sessions create opportunities for attendees to organically meet individuals with similar interests, goals, and career paths.

In addition to these networking opportunities, mentorship remains a foundational element of the APSA Annual Meeting. Workshops have focused on foundational skills, such as “Effective Communication in Research,” “Mentorship Strategies for Emerging Scientists,” “Effective Mentorship: Building Successful Relationships,” “Keys to Successful Mentoring Relationships,” and “Mentorship and Leadership in Academic Medicine,” emphasizing the bidirectional responsibilities of mentees and mentors. Workshops have also addressed mentorship in specific contexts, such as “Mentorship in Translational Medicine” and “Effective Mentorship in Biomedical Sciences.”

In 2023, the AAP, led by Dianna Milewicz, MD, PhD, formed the Physician-Scientist Trainee Network (PSTN), a year-round coaching initiative. The PSTN program pairs APSA members with AAP and ASCI members to build enduring, long-term mentoring relationships that extend beyond the confines of a single, yearly meeting. The AAP and ASCI’s support has provided an unparalleled platform for intergenerational mentorship.

Together, these offerings reflect APSA’s enduring commitment to community building as a pillar of its mission to support the next generation of physician-scientists.

### Accessibility and advocacy.

The APSA is committed to fostering and expanding an inclusive physician-scientist workforce (3–5). At the Joint Meeting, the APSA hosts a variety of panels and workshops to help attendees develop and cultivate practical skills to create a more diverse and inclusive health care system. Beginning in 2005 with the seminar “Advocating for Diversity in Science,” these sessions have emphasized the importance of inclusive research and medical environments, starting at the trainee level. Similar themes have continued annually, including “Promoting Inclusivity in Scientific Research,” “Addressing Women and Underrepresented Minorities (URM) Disparities in Academia,” “Policies and Ethics of Medical Innovation,” and “Transforming Workplace Culture with Collective Courage.” Additional panels, including “Reproductive Politics: Critical Perspectives on Care and Advocacy” and “Effects of the Current Policy Landscape on Health Equity,” have provided valuable opportunities for attendees to learn about the significant effects they can have in shaping policies and driving change within their institutions and communities. Together, these sessions reflect a sustained and expanding commitment to accessibility, inclusivity, and social responsibility in the physician-scientist community.

### Scientific discoveries.

Finally, biomedical innovation has been the intellectual foundation of the AAP/ASCI/APSA Joint Meeting since its inception. Every year, the meeting features keynote addresses, plenary sessions, invited talks, and trainee presentations that highlight major advances in biomedical research and clinical science. Notably, in addition to the “Interviewing an Icon” session described above, the Lasker Foundation sponsors a laureate to deliver a keynote address, further augmenting the caliber of programming provided at the meeting.

## Attendee growth and reach

**Figure 1:**
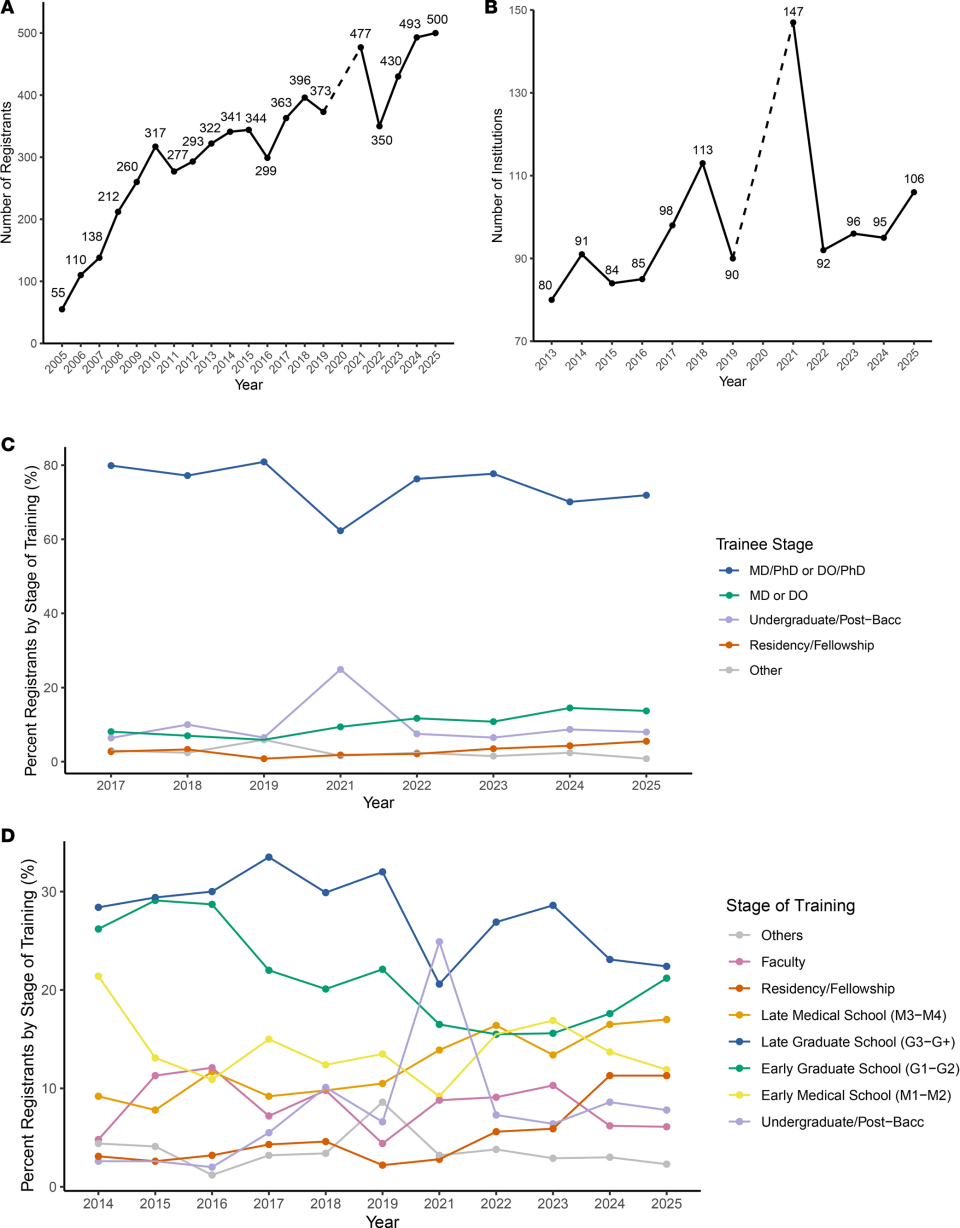
APSA attendee distribution by year. (**A**) The number of APSA attendees at the Joint Meeting from 2005–2025. (**B**) The number of institutions, both nationally and globally, represented at the meeting as APSA-affiliated registrants. The APSA began collecting data on institutional affiliations in 2013. (**C**) Proportion of participants by degree type. The APSA began collecting data on degree type in 2017. (**D**) Proportion of participant career stage. The APSA began collecting data on training stage in 2014. Due to the COVID-19 pandemic, the meeting in 2020 was cancelled, and the meeting in 2021 was conducted virtually.

Participation has grown substantially since 2005, when the meeting welcomed 55 APSA participants. In 2025, a record of 500 APSA-affiliated participants attended, accounting for almost half of all participants at the AAP/ASCI/APSA Joint Meeting (Figure 1A). The number of participating institutions has also grown from 80 unique institutions in 2013 to over 100 in 2025, demonstrating a trend of greater institutional involvement over time (Figure 1B). In addition, the global reach of the APSA has expanded, with registrants hailing from institutions in 13 countries besides the United States, including Brazil, Canada, China, Finland, France, India, Kazakhstan, Pakistan, Singapore, Slovakia, South Korea, Switzerland, and the United Kingdom. This international engagement aligns with APSA’s commitment to global collaboration through its participation in the International Consortium of Clinician Scientist Trainee Organizations. During this 20-year time frame, the annual meeting was cancelled only once, in 2020 due to the COVID-19 pandemic, with a virtual meeting following in 2021. All other conferences have been in person in Chicago. Over time, the conference has drawn a growing audience beyond dual-degree students, including undergraduate and postbaccalaureate students, non–dual-degree MD/DO students, residents, fellows, and more (Figure 1C). Participants span all stages of the physician-scientist trainee path, from undergraduate students to early-career faculty (Figure 1D). Out of 4,226 unique registrants, 1,042 registered to attend the Joint Meeting more than once (24.7%). The average yearly return rate for an individual registering in the following year was 18.2%. APSA-affiliated participants have grown in number and across career stages to gain mentorship, community, and professional development.

**Figure 2:**
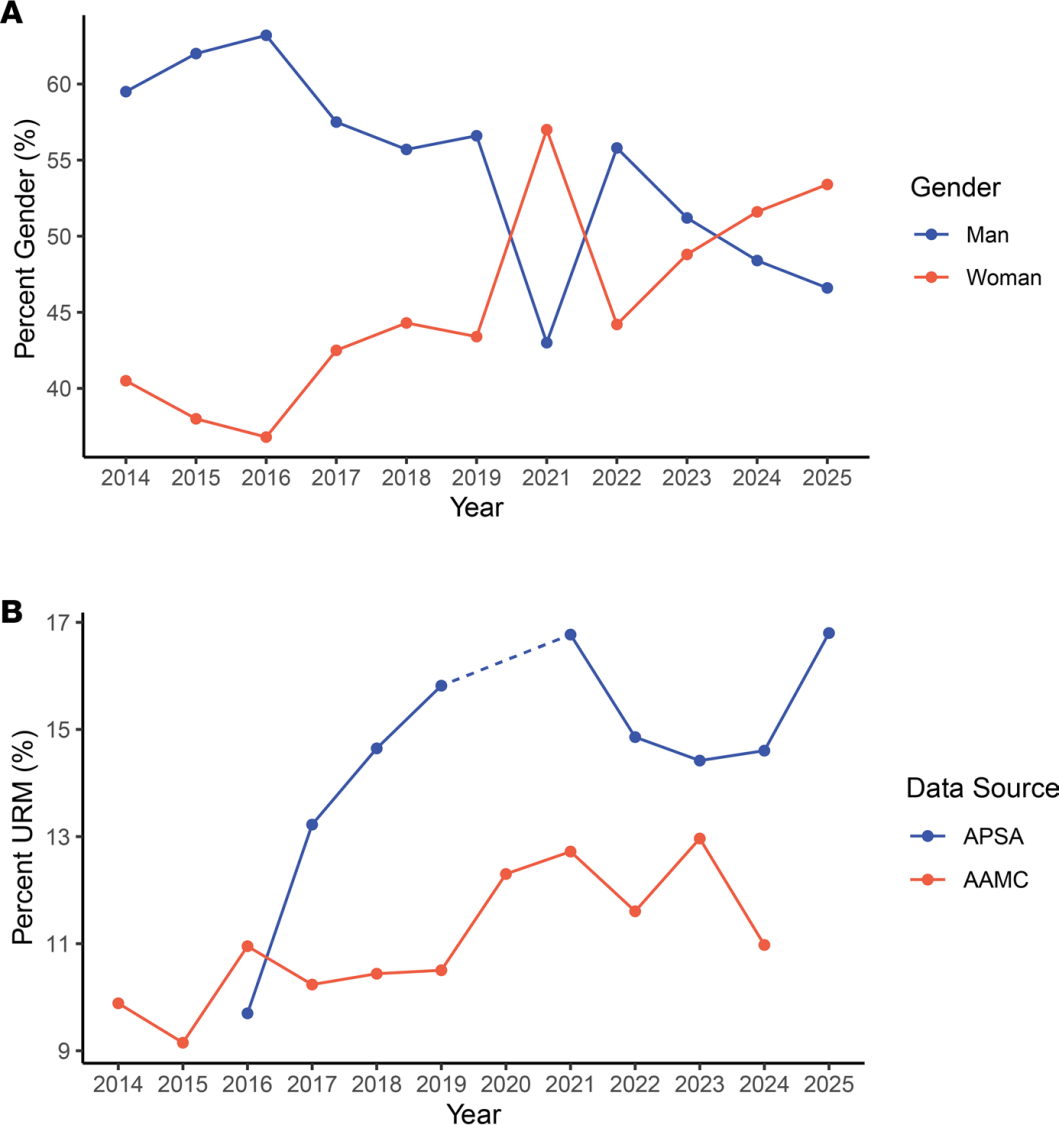
APSA participant demographics per year. (**A**) Participant representation based on gender. Of registrants, 154 declined to respond, 10 responded with “non-binary/non-conforming,” 4 “genderqueer,” and 9 “not listed/other.” (**B**) Percentage of APSA meeting participants from groups considered to be underrepresented in medicine (URM) compared to MD-PhD matriculation data from the Association of American Medical Colleges (AAMC). The AAMC made demographic information available beginning in 2014 and the APSA began collecting registrant gender in 2014 and race and ethnicity information in 2016. AAMC FACTS tables for 2025 were not available at the time of analysis. AAMC data were drawn from AAMC FACTS Table B9: https://www.aamc.org/data-reports/students-residents/report/facts Both the APSA and the AAMC define URM as individuals identifying as American Indian or Alaska Native, Black or African American, Hispanic, Latino, or of Spanish Origin, or Native Hawaiian or Other Pacific Islander (7).

The APSA is committed to creating an inclusive environment at the Joint Meeting. Efforts to achieve this goal have included (a) invitation of speakers from a wide range of backgrounds across the spectrum of gender, race, ethnicity, specialty, and career sector; (b) curation of sessions that promote equitable opportunities in training and health care; and (c) creation of multiple opportunities throughout the conference for individuals to network and identify mentors with similar interests. The success of these efforts is reflected in the increasing diversity of APSA participants at the meeting. In particular, there has been a substantial increase in the number of female participants (Figure 2A) and individuals from URM backgrounds to a level that is greater than the national proportion of URM students matriculating into US MD-PhD programs, based on data from the Association of American Medical Colleges (AAMC) (Figure 2B).

**Figure 3:**
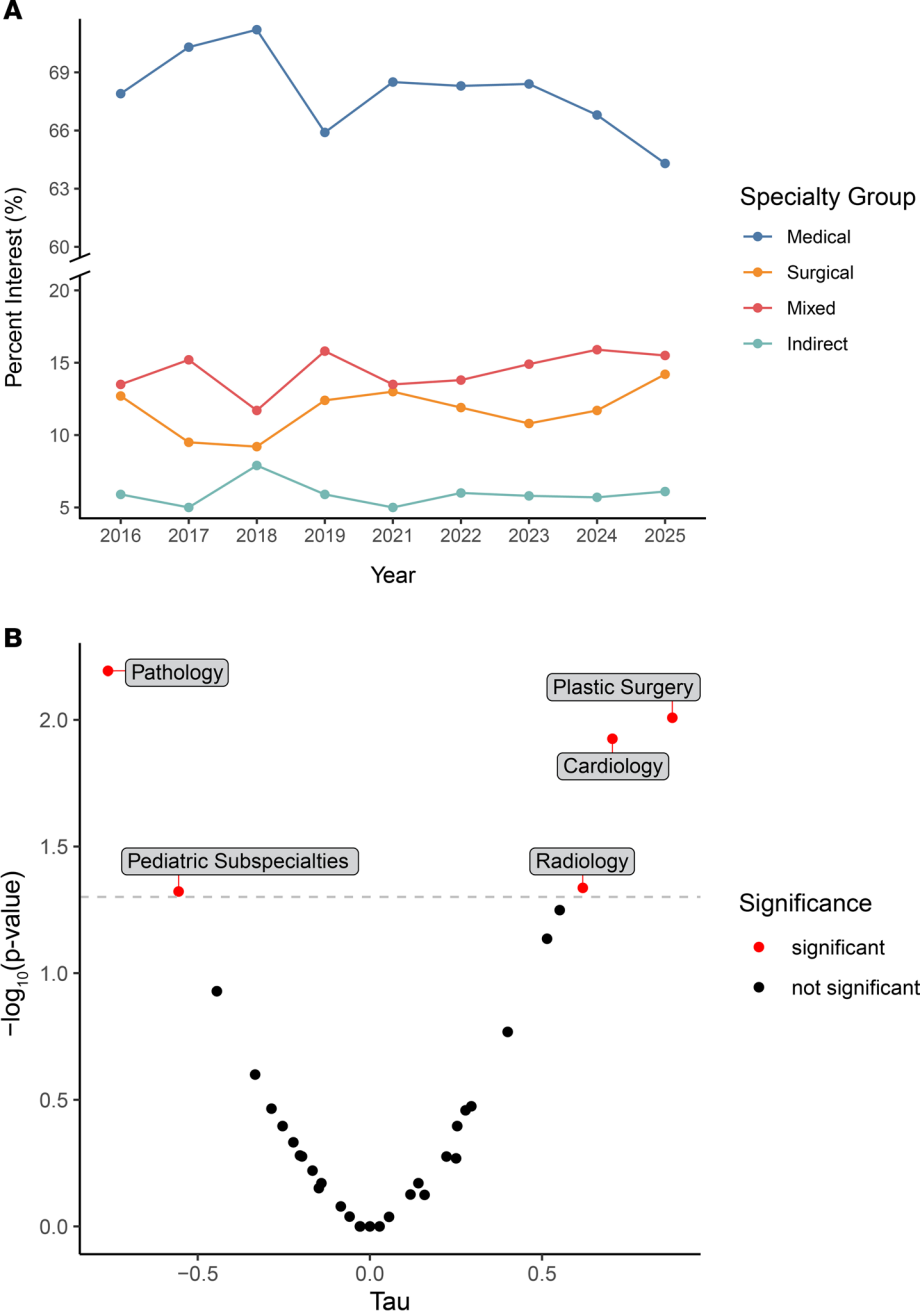
Specialty interests reported by participants per year. (**A**) Residency interests grouped by specialty type. The *y* axis has been split to better display the higher proportion of medical specialty interest. (**B**) Changes in specialty interests from 2016–2025 performed using the Mann-Kendall test to detect increasing or decreasing trends in time series data. A positive Tau score is associated with a positive correlation (increased interest), while a negative Tau score is associated with a negative correlation (decreased interest). Points in red denote a significant increase or decrease in interest over time. The APSA began collecting participant data on specialty interests in 2016.

In addition to expanding participant demographics, we have seen broader interests in medical specialties. We found decreased interest in medical specialties (internal medicine, family medicine, pediatrics, neurology, child neurology, psychiatry, physical medicine and rehabilitation, radiation oncology, and medical genetics); increased interest in surgical specialties (general surgery, neurological surgery, orthopedic surgery, plastic surgery, vascular surgery, and thoracic surgery) and mixed specialties (obstetrics and gynecology, ophthalmology, otolaryngology, urology, anesthesiology, dermatology, emergency medicine, and interventional radiology); and steady interest in indirect patient care (pathology and radiology) (Figure 3A). Within these categories, specialties including cardiology, plastic surgery, and radiology have seen a significant increase in interest, while pediatric subspecialties and pathology have seen a decline (Figure 3B).

The APSA has actively worked to create an environment that fosters a wide range of perspectives and broadens access to opportunities within the physician-scientist community. This expansion of participants is further made possible by the support of the numerous travel awards donated by our partners over time.

## The 20th Annual Meeting: a defining moment

**Figure 4:**
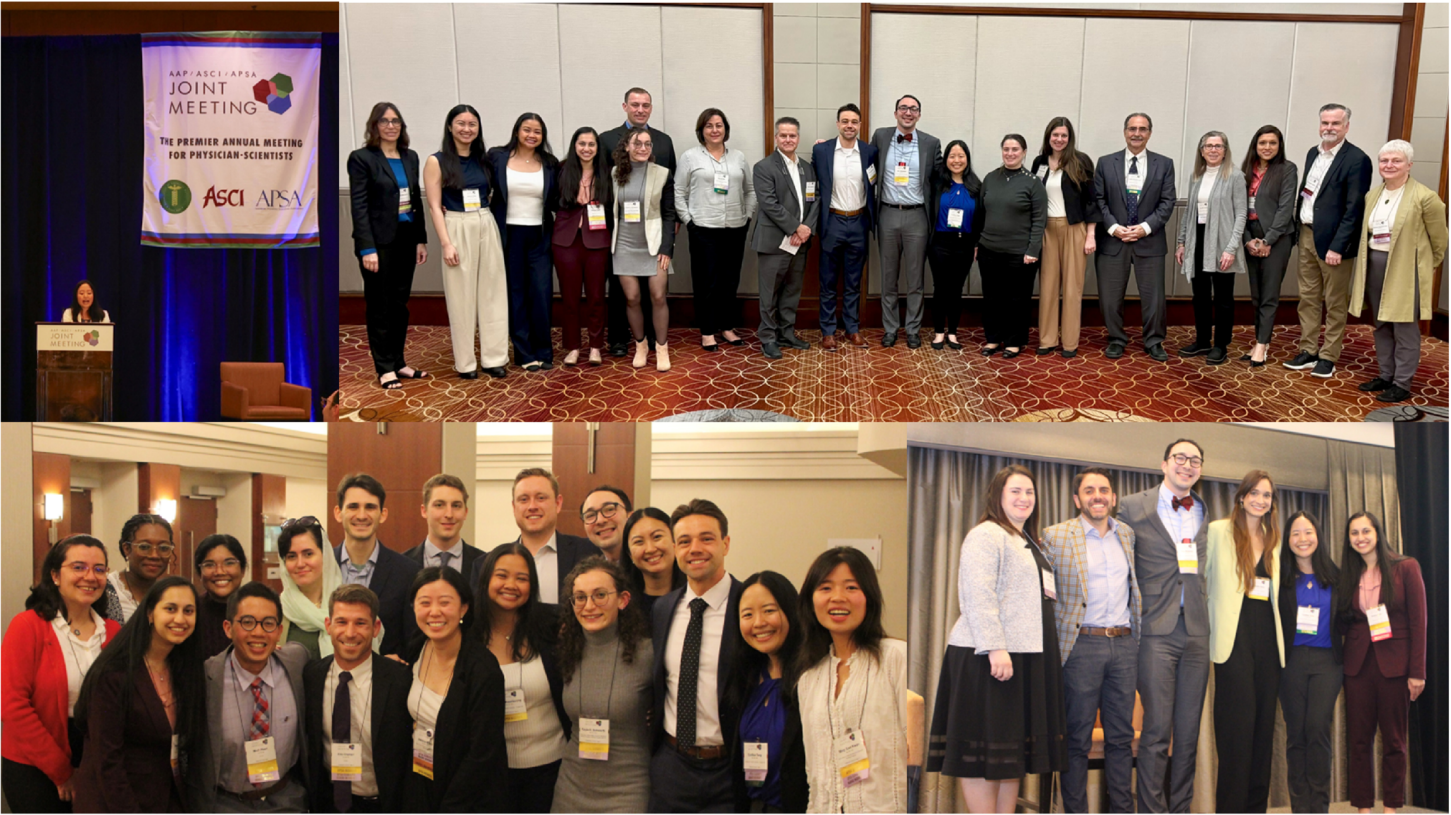
The APSA celebrates its 20th Annual Meeting at the 2025 AAP/ASCI/APSA Joint Meeting. Top left, Cynthia Tang, PhD, gives the Presidential Address. Top Right, incoming and outgoing APSA Board of Directors. Bottom left: 2024–2025 APSA Executive Council. Bottom right: Past and future APSA Presidents.

The 20th Annual Meeting in 2025 was a landmark event, reflecting on APSA’s past achievements while charting the future of physician-scientist training (Figure 4), with themes emphasizing innovation, collaboration, and the evolving role of physician-scientists in the biomedical landscape. This meeting started with an inspiring live interview with Peggy Hamburg, MD, Co-President of the InterAcademy Partnership, former Commissioner of the US Food and Drug Administration, and former Commissioner of the New York City Department of Health, among many other roles, which was hosted in collaboration with the Lasker Foundation. This session was followed by an innovative look to the future of biomedicine and the integration of artificial intelligence, with Vivian Lee, MD, and the APSA Presidential Address, which underscored the organization’s role in improving accessibility for future and current physician-scientists in training as well as its vision for the future. Svetlana Mojsov, PhD, gave the 2025 APSA~Lasker Laureate Lecture, “Chemistry Matters: From a Putative Peptide to Effective Medicines for Diabetes and Obesity.” This was in addition to a weekend full of breakthrough scientific talks, with APSA-hosted sessions including “Career Opportunities in Biotech and Pharmaceuticals” with Larry Schlesinger, MD, and Julie Louise Gerberding, MD, MPH; “Transforming Workplace Culture with Collective Courage” with Pringl Miller, MD; “Closing the Gaps Between Discovery, Delivery, and Policy” with Susan Cheng, MD, MMSc, MPH, and Elbert Huang, MD, MPH, FACP; “Launching Your Physician-Scientist Journey: Strategic Moves in Residency and Fellowship” with Dineo Khabele, MD, and Ebru Erbay, MD, PhD; and “Reproductive Politics: Critical Perspectives on Care and Advocacy” with Ashish Premkumar, MD, PhD. In response to the increasing demand for near-peer mentorship, the APSA hosted its first “Near Peer Mentorship Hour,” with trainee volunteers from across the nation sharing their experiences and advice for navigating the so-called hidden curriculum. Finally, the APSA recognized Beatrice Renault, PhD, Chief Scientific Officer of the Albert and Mary Lasker Foundation, as the 2025 Founder’s Award recipient and Eli Wisdom, APSA’s 2024–2025 Vice President, as the 2025 President’s Service Award recipient at the APSA Awards Dinner.

This year, over 60 promising trainees were supported by travel awards given by society donors, including the AAP, ASCI, NIH, BWF, American Society for Investigative Pathology, Foundation for Anesthesia Education and Research, American Society of Nephrology, Society for Academic Emergency Medicine, American Association of Immunologists, Association of Medical School Pediatric Department Chairs, and Texas Biomedical Research Institute. This support contributed to a record number of APSA-affiliated attendees at the meeting, representing 106 global institutions. Moreover, 34 residency programs participated in the Residency Luncheon, and 2 MD-PhD programs participated in a pilot program aimed at customizing the luncheon for early-stage trainees applying to dual-degree programs. Over 80 AAP and ASCI members volunteered to mentor more than 270 trainees as part of the PSTN program. The success of this milestone meeting reflects both the need for and strength of the physician-scientist community.

## The strength of collaboration: AAP and ASCI

The Joint Meeting serves not only as a venue for presenting science but also as a springboard for career trajectories, fostering connections that span the physician-scientist career path. The extensive growth and expansion of APSA’s attendance at the Joint Meeting would not be possible without APSA’s long-standing collaboration with the AAP and ASCI. This annual gathering of the nation’s leading physician-scientists provides trainees the opportunity to engage with role models and gain career and training insights across academic medicine, government, and industry. The AAP and ASCI have also provided mentorship through the formalized coaching programs and travel awards discussed above. In addition, the ASCI provides essential funding to support APSA’s regional meetings, and APSA leaders serves as consulting members on the ASCI Physician-Scientist Development Committee. APSA’s partnerships with the AAP and ASCI have been instrumental in enhancing physician-scientist training and career development. Leaders from the AAP, ASCI, and APSA have consistently highlighted the transformative effect of these partnerships, underscoring the importance of a collaborative approach in shaping the future of the physician-scientist workforce.

## The future of the APSA and maintaining the physician-scientist pipeline

As the APSA looks to the next decade, several challenges and opportunities lie ahead. Physician-scientists often serve as the pioneers and leaders of biomedical discoveries; however, increased funding and career instability threaten the sustainability of this career path. Leaders in the physician-scientist community must continue to address current and emerging barriers to maintaining a robust physician-scientist pipeline (6). Invested parties must continue the to strengthen and expand collaborative efforts, such as the joint initiatives between the AAP, ASCI, and APSA, to ensure an accessible and sustainable pipeline. Finally, the continued success of the APSA will rely on sustained advocacy, strategic collaborations, and a commitment to nurturing the next generation of physician-scientists.

## Conclusions

The 20th APSA Annual Meeting serves as both a reflection on past achievements and a call to action for the future. The organization’s contributions to physician-scientist training, career development, and advocacy have shaped countless careers and advanced biomedical research. Continued collaboration with the AAP and ASCI will be crucial in ensuring that physician-scientists remain at the forefront of medical innovation. As we enter the next decade of APSA meetings, we invite all invested parties to join in supporting and strengthening the physician-scientist pipeline for years to come through financial donations and/or by volunteering with leadership, committees, and mentorship programs. During this next chapter, we are optimistic that we will see a growing, thriving, and diverse community that is accessible to all.

## Author contributions

CYT is the 2024–2025 Past President of the APSA and conceptualized and wrote the manuscript. ADW is the 2023–2024 Past President of the APSA and edited the manuscript. DCB is a past Executive Council member of the APSA and analyzed the data, generated the figures, and edited the manuscript.

## Version 1 09/09/2025

Electronic publication
